# Do changes in income and social networks influence self-rated oral health trajectories among civil servants in Brazil? Evidence from the longitudinal Pró-Saúde study

**DOI:** 10.1186/s12903-022-02191-5

**Published:** 2022-04-29

**Authors:** Mario Vianna Vettore, Mauro Henrique Nogueira Guimarães Abreu, Suellen da Rocha Mendes, Eduardo Faerstein

**Affiliations:** 1 Department of Health and Nursing Sciences, University of Agder (UiA), Campus Kristiansand, Universitetsveien 25, 4630 Kristiansand, Norway; 2 Department of Social and Preventive Dentistry, School of Dentistry, Federal University of Minas Gerais, Belo Horizonte, MG Brazil; 3 Department of Epidemiology, Institute of Social Medicine, State University of Rio de Janeiro, Rio de Janeiro, Brazil

**Keywords:** Social determinants of health, Income, Social networks, Oral health, Longitudinal studies

## Abstract

**Background:**

Social factors are important determinants of health. However, evidence from longitudinal studies on the possible role of changes in socioeconomic circumstances on adult’s oral health is scarce. This study aimed to test whether changes in income and changes in social networks of family members and friends were associated with trajectories of self-rated oral health (SROH) among adults over a 13-year period.

**Methods:**

A prospective cohort study (Pro-Saude Study) was conducted involving non-faculty civil servants at university campi in Rio de Janeiro, Brazil. Individual data was collected through self-completed questionnaires in four waves (1999, 2001, 2007 and 2012). SROH trajectories between 2001 and 2012 were “Good-stable SROH”, “Changed SROH”, “Poor-stable SROH”. *Per capita* family income and social networks of family members and friends data obtained in 1999 and 2012 were grouped into “High stable”, “Increase”, “Decrease”, “Low stable”. Ordinal logistic regression using complete data of 2118 participants was used to estimate odds ratio (OR) and 95% CIs of changes in income and changes in social networks with SROH trajectories, adjusted for age, sex, skin colour and marital status.

**Results:**

Participants in the low income-stable and small social networks-stable groups showed 2.44 (95% CI 1.68–3.55) and 1.98 (95% CI 1.38–2.85) higher odds for worst trajectory of SRHO than those in the respective high-stable groups. Those in the decrease income group and decrease social networks group were 78% (95% CI 1.25–2.54) and 58% (95% CI 1.07–2.34) more likely to worst trajectory of SRHO than those in the high income-stable and high social networks-stable groups.

**Conclusions:**

Adults reporting low income and low social networks of family members and friends over 13 years and those with income and social networks decrease during the study period were at higher risk of having worsened their self-rated oral health.

## Background

The possible influence of social determinants on oral health acknowledges that individuals who persistently experience social disadvantage or economic obstacles have worse oral health than those from more advantaged socioeconomic groups. Oral health disparities refer to the social patterning of health resulted from the uneven distribution of diseases across different social strata in a population [1]. There is voluminous literature demonstrating a consistent stepwise relationship between socioeconomic status and the severity of oral conditions, suggesting oral health disparities are socially patterned [2, 3]. However, oral health inequalities are predominantly supported by cross-sectional studies and evidence from cohort studies is increasing more recently [2].

The social mobility hypothesis combines sensitive periods and accumulation hypotheses, depending on whether individuals remain or change between different categories of the socioeconomic strata during the life course [4]. Longitudinal analyses have shown that early life circumstances and social mobility can negatively influence oral health during adulthood [5–9]. Children who grew up in high socioeconomic status families, assessed through parent’s occupational status or family income had lower likelihood to have unsound tooth (a filled tooth with dental caries or a decayed or missing tooth), dental caries, periodontal disease, and tooth loss in adulthood than those who experienced a decline in their social and economic circumstances over time (downward social mobility) [5–9]. Remaining in less advantageous social groups from birth to adulthood was also related to poor oral health when compared with those who persistently were in the higher social groups [6–9]. Few studies investigated the role of social mobility on oral health during adulthood [8–10]. Adults who experienced downward social mobility showed higher odds of worse self-rated oral health and were less likely to retain functional dentition than those who remained with high income [8–10].

Oral health outcomes have been associated with social networks among different age groups [11–14]. Social networks is a broad term referring to social ties originated from the structural social arrangements that shape the resources available to individuals, influencing their behavioural and emotional responses [15]. The concept of social networks adopted in this study refers to the ‘web’ of social relationships with whom the individual maintains close social bond and mutual trust [16]. This definition acknowledges the importance of intimate contacts surrounding the individual in the determination of health status, including the number of social ties with friends and relatives [16]. Social networks assessed considering the involvement with different social groups were associated with self-reported number of remaining teeth in Japanese elderly [11, 12]. Also, better oral health-related quality of life and better dental status were predicted by larger support network size and greater social support lower among and adolescents and post-partum women [13, 14].

Preliminary analyses of a prospective cohort study involving civil servants in Rio de Janeiro, Brazil, showed that lower social position and weak social ties at baseline were associated with tooth loss and self-rated oral health after 13 years of follow-up [17]. Evidence on the association between social mobility during adulthood and oral health is scarce [8–10]. In addition, previous studies on this topic did not consider changes in oral health measures as outcomes [8–10]. As far as the authors are aware, the possible relationship between changes in social networks and adult’s oral health trajectories was not examined prospectively [11–14].

The aim of the present study was to investigate the influence of changes in income and changes in the number of family members and friends in social networks of on self-rated oral health trajectories in adults over a 13-year follow up period. We hypothesised that adults experiencing a downward social mobility and a decrease in the number of family members and friends in the social networks over the study period are more likely to report a worsening of self-rated oral health than those with stable high income and large stable number of family members and friends in the social networks. It was also conjectured that a worsening of self-rated oral health was associated with low stable income and small stable number of family members and friends in the social networks over the 13 years period among adults.

## Methods

The Pro-Saude study is a prospective longitudinal study conducted at several university campuses in the State of Rio de Janeiro, Brazil, involving non-faculty civil servants. All technical and administrative permanent staff members were invited for the study. The exclusion criteria were current non-medical leave of absence and working relocation to another institution.

Trained personnel using self-administered multidimensional questionnaires collected data at participants’ workplaces up [18]. Pro-Saude study data collection was carried out in 1999, 2001, 2007 and 2012, representing waves 1, 2, 3 and 4, respectively. Wave 1 of data collection was conducted in 1999 (N = 4030; response rate = 90.4%). Follow-up data collection was carried out in 2001, 2007 and 2012; characterizing waves 2, 3 and 4, respectively. Wave 2 and wave 4 included 3574 (response rate = 80.2%) and 3058 participants (response rate = 68.6%), characterising a 13-year interval period. Data from wave 3 was not relevant for the present study since neither social networks nor self-rated oral health were evaluated in this wave. Participants with incomplete data were excluded from the analysis resulting in a final analytic sample of 2118 adults (69.3% of wave 4).

### Self-rated oral health

Self-rated oral health was assessed by the question “In general, how would you rate your oral health status?”. The following response options were used: very good, good, fair, poor/bad, very poor/very bad) [19, 20]. The outcome was a three-point categorical variable representing SRHO trajectories developed by combining self-rated oral health (SROH) measures collected in waves 2 and 4 as follows: “Good-stable SROH”: very good/good/fair at waves 2 and 4; “Changed SROH”: poor/very poor at wave 2 and very good/good/fair at wave 4 or very good/good/fair at wave 2 and poor/very poor at wave 4; “Poor-stable SROH”: poor/very poor at waves 2 and 4.

### Income changes

Changes in *per capita* monthly income and changes in social networks of family members and friends were the main exposures. The *per capita* monthly income was assessed according to the total earnings of the residents in the household and categorized as < 3 Brazilian minimal wages [BMW]; 3–6 BMW; > 6 BMW. One BMW was US$57.17 and US$303.42 in 1999 (wave 1) and 2012 (wave 4), respectively. *Per capita* monthly income was then categorised as low income (≤ 3 BMW) and high (> 3 BMW) as this seems a reasonable cut-off between lower and upper social classes in Brazil. The two upper income categories used in this study were considered soundly akin according to previous research since social inequalities in health follow a ‘bottom inequity’ pattern in Brazil [7, 10]. These two categories of *per capita* monthly income were used to generate the four groups of changes in income: “high income-stable”: high income at waves 1 and 4; “increase income”: low income at wave 1 and high income at wave 4; “decrease income”: high income at wave 1 and low income at wave 4; “low income-stable”: low income at waves 1 and 4.

### Social networks changes

Number of family members and friends in social networks were measured in waves 1 and 4 using the same questions utilised in the Whitehall study [21]: “How many family members/friends do you feel comfortable with and can talk about almost everything?” [22] Participants were classified into four groups: “Large social networks stable”: ≥ 3 family members and friends at waves 1 and 4; “Increased social networks size”: ≤ 2 at wave 1 and ≥ 3 at wave 4; “Decreased social networks size”: ≥ 3 at wave 1 and at ≤ 2 at wave 4; “Small social networks stable”: ≤ 2 family members and friends at waves 1 and 4. Large and small social networks were represented by greater and lower number of social relationships between the participant and their family members and friends, respectively.

### Covariates

Demographic and socioeconomic characteristics assessed at wave 1 (1999) were analysed as confounders on the influence of income changes and social network changes on SROH trajectories according to a proposed theoretical model previously published [17]. The covariates included age, sex (male; female), self-reported skin colour (white; brown/pardo; black; other), marital status (single; married; divorced; widowed), and educational attainment (≤ 10 years; 11–15 years; ≥ 16 years).

### Pilot study

A pilot study involving 1120 temporary civil servants at the same university campuses who were not eligible to participate in the main study was conducted to assess the temporal reliability of the instruments. Kappa coefficients and intra-class correlation (ICC) coefficients were used to assess reliability for the test–retest categorical responses to the SROH question and for the number of social networks of family members and friends, respectively. SROH showed very good test–retest reliability (Kappa = 0.80; 95% CI = 0.69–0.89) [20]. ICC coefficients for social networks of family members and friends were 0.70 (95% CI = 0.62–0.77) and 0.77 (95% CI = 0.70–0.82), indicating moderate and good reliability, respectively [23].

### Statistical analysis

All variables were compared between participants with missing data (N = 940) and those with complete data (N = 2118) using *t*-test and Pearson Chi-square test for continuous and categorical variables, respectively. The distribution of demographic and socioeconomic characteristics, income groups and social networks groups were presented according to SROH trajectories groups using means (SD) and proportions for continuous and categorical variables, respectively.

Ordinal logistic regression was carried out to assess the influence of changes in income and changes in the number of family members and friends in social networks of on self-rated oral health trajectories over the study period. Odds ratios (ORs) and 95% confidence intervals (CIs) were estimated for the independent variables using the *logit* function. “Good-stable SROH” was the reference category for the outcome variable. The reference category for change in income and change in social networks of family members and friends were “high income-stable” and “large social networks stable”, respectively. Substantial correlations using Spearman’s coefficients were observed between income in 1999 and educational attainment (ρ = 0.570), income in 2012 and educational attainment (ρ = 0.543), number of social networks in 1999 and educational attainment (ρ = 0.164), and number of social networks in 2012 and educational attainment (ρ = 0.183). Four statistical models were tested. Model 1 assessed the crude association of income groups and social networks of family members and friends groups with SROH trajectories. In Model 2, income groups and social networks variables were adjusted for each other. Model 3 included demographic variables (age, gender and self-reported skin colour). Socioeconomic variable marital status was inserted in Model 4. Educational attainment was not considered in the regression models due to collinearity with the exposures. All analyses were carried out using IBM SPSS Statistics 25.0 (SPSS, Chicago, IL, USA).

### Ethic aspects

The present research was approved by the Research Ethics Committee of the Institute of Social Medicine, State of University of Rio de Janeiro (CAAE 0041.0.259.000-11). Informed consent was obtained before data collection.

## Results

Demographic and socioeconomic characteristics, income groups, number of family members and friends in social network, and self-reported oral health of excluded participants due to missing data (N = 940) and those with complete data (analysed sample) (N = 2118) are presented in Table 1. Participants with complete data were younger, experienced greater educational attainment, and had higher income than those with missing data. Number of family members and friends in the social networks and self-reported oral health did not differ between participants with and without complete data.

**Table 1:** Demographic and socioeconomic characteristics, income groups, number of family members and friends in the social networks, and self-rated oral health between excluded participants due to missing data and those with complete data

Variable (year)	Participants with missing data (N = 940)	Final analytic sample (N = 2118)
*Demographic characteristics (1999)*		
Age (years), Mean (SD)*	41.6 (9.1)	39.1 (7.8)
Sex, N (%)*		
Male	437 (46.5)	903 (42.6)
Female	503 (53.5)	1215 (57.4)
Skin colour, N (%)*		
White	438 (46.6)	1101 (52.0)
Brown/pardo	315 (33.5)	640 (30.2)
Black	155 (16.5)	323 (15.3)
Other	32 (3.4)	54 (2.5)
*Socioeconomic characteristics (1999)*		
Marital status, N (%)		
Single	198 (21.0)	443 (20.9)
Married	548 (58.3)	1313 (62.1)
Divorced	165 (17.6)	300 (14.2)
Widowed	29 (3.1)	59 (2.8)
Educational attainment, N (%)*		
≤ 10 years	309 (32.9)	440 (20.8)
11–15 years	325 (34.6)	802 (37.9)
≥ 16 years	306 (32.5)	876 (41.3)
Per capita monthly income, N (%)*		
< 3 BMW	257 (27.4)	588 (27.8)
3–6 BMW	361 (38.4)	804 (38.0)
> 6 BMW	322 (34.2)	726 (34.3)
Income groups, N (%) (1999–2012)*		
High income-stable	265 (28.2)	754 (35.6)
Increase income	50 (5.3)	78 (3.7)
Decrease income	211 (22.5)	764 (30.1)
Low income-stable	414(44.0)	522 (24.6)
Social networks groups (1999–2012)		
Number of family members/friends in the social networks, N (%)		
Large social networks stable	471 (50.1)	1314 (62.0)
Increased social networks	169 (18.0)	299 (14.1)
Decreased social networks	108 (11.5)	249 (11.8)
Small social networks stable	192 (20.4)	256 (12.1)
*Self-rated oral health, N (%) (1999–2012)*		
Good-stable	837 (89.0)	1813 (85.6)
Changed	81 (8.6)	230 (10.9)
Poor-stable	22 (2.4)	75 (3.5)

Pro-Saude study, Rio de Janeiro, Brazil, 1999–2012

*BMW* Brazilian Minimal Wage, *P < 0.05

The mean age of the sample at wave 1 data collection was 39.1 years, ranging from 22 to 67 years. Participants were predominantly females (57.4%). Of the sample, 41.3% had education for at least 16 years and 56.2% had *per capita* family income up to six Brazilian minimum wages. Most participants experienced a  high stable income (35.6%) and large stable number of family members and friends in the social networks (62%) during the study period. The majority of the sample (85.6%) was in the Good SROH group (Table 1).

Table 2 presents the distribution of demographic and socioeconomic characteristics, income groups and number of family members and friends in the social networks between SROH trajectories groups. Younger participants, females, those with greater education attainment, and in the high income-stable and high social networks-stable groups prevailed in the good-stable SROH group than their counterparts.

**Table 2:** Distribution of demographic and socioeconomic characteristics, income groups, social networks groups according to self-rated oral health trajectories groups

Variable	Good-stable SROH	Changed SROH	Poor-stable SROH
*Demographic characteristics (1999)*			
Age (years), Mean (SD)	38.8 (7.8)	40.7 (7.7)	41.0 (7.5)
Sex, N (%)			
Male	750 (41.4)	119 (51.7)	34 (45.3)
Female	1063 (58.6)	111 (48.3)	41 (54.7)
Self-reported skin colour			
White	962 (54.7)	87 (39.5)	27 (38.6)
Brown/Pardo	444 (25.2)	75 (34.1)	19 (27.1)
Black	345 (19.6)	58 (26.4)	23 (32.9)
Other	8 (0.5)	0 (0.0)	1 (1.4)
*Socioeconomic characteristics (1999)*			
Marital status			
Single	379 (21.3)	38 (17.0)	8 (11.0)
Married	1109 (62.3)	146 (65.2)	46 (63.0)
Divorced	252 (14.1)	29 (12.9)	14 (19.2)
Widowed	41 (2.3)	11 (4.9)	5 (6.8)
Educational attaiment, N (%)			
≤ 10 years	327 (18.0)	84 (36.5)	29 (38.7)
11–15 years	673 (37.2)	100 (43.5)	29 (38.7)
≥ 16 years	813 (44.8)	46 (20.0)	17 (22.6)
Income groups (1999–2012), N (%)			
High income-stable	664 (38.4)	39 (17.3)	19 (26.0)
Increase income	61 (3.5)	11 (4.9)	3 (4.1)
Decrease income	623 (36.0)	84 (37.3)	25 (34.2)
Low income-stable	383 (22.1)	91 (40.4)	26 (35.7)
Number of family members/friends in the social networks (1999–2012), N (%)			
Large social networks stable	1168 (64.4)	119 (51.7)	27 (36.0)
Increased social networks	247 (13.6)	37 (16.2)	15 (20.0)
Decreased social networks	202 (11.2)	30 (13.0)	17 (22.7)
Small social networks stable	196 (10.8)	44 (19.1)	16 (21.3)

Pro-Saude study, Rio de Janeiro, Brazil, 1999–2012

*SROH* self-rated oral health

Ordinal logistic regression models estimated the association of income groups, number of family members and friends in the social networks with SROH trajectory groups (Table 3). In the crude analysis (Model 1), all categories of income groups and social networks of family members and friends predicted worse SROH over a 13-year period. Income groups and social networks of family members and friends remained associated with worse SROH after mutual adjustment (Model 2) and for demographics (Model 3). In the final model (Model 4), adults in the increase income, decrease income and low income-stable groups showed 2.65 (95% CI 1.17–4.38), 1.78 (95% CI 1.25–2.54) and 2.44 (95% CI 1.68–3.55) higher odds of worse SROH than those in the high income-stable group. In addition, adults with decrease social networks and small stable social networks of family members and friend were 58% (OR = 1.58, 95% CI 1.07–2.34) and 98% (OR = 1.98, 95% CI 1.38–2.85) more likely to report worse SROH than those in the large stable social networks of family members group.

**Table 3:** Ordinal logistic regression analysis of income groups, social networks of relatives, social networks of friends and self-rated oral health trajectory groups

Variable	Model 1	Model 2	Model 3	Model 4
	OR (95% CI)	OR (95% CI)	OR (95% CI)	OR (95% CI)
Income groups				
High income-stable	1	1	1	1
Increase income	2.58 (1.37–4.89)**	2.38 (1.25–4.51)**	2.24 (1.17–4.31)*	2.65 (1.17–4.38)*
Decrease income	1.98 (1.42–2.77)**	1.89 (1.35–2.65)**	1.80 (1.27–2.56)**	1.78 (1.25–2.54)**
Low income-stable	3.43 (2.45–4.81)**	3.01 (2.13–4.24)**	2.44 (1.69–3.85)**	2.44 (1.68–3.55)**
Number of family members/friends in the social networks				
Large social networks stable	1	1	1	1
Increased social networks	1.71 (1.21–2.41)**	1.48 (1.04–2.11)*	1.43 (0.99–2.06)	1.29 (0.89–1.89)
Decreased social networks	1.92 (1.34–2.75) **	1.71 (0.58–1.38)**	1.61 (1.10–2.36)*	1.58 (1.07–2.34)*
Small social networks stable	2.47 (1.75–3.45)**	2.12 (1.49–3.00)**	1.95 (1.36–2.80)**	1.98 (1.38–2.85)**

Pro-Saude study, Rio de Janeiro, Brazil, 1999–2012

Model 1: crude association of income groups and social networks of family members and friends with self-rated oral health trajectory groups

Model 2: mutually adjusted for income groups and social networks of family members and friends

Model 3: Model 2+ adjusted for age, gender and self-reported skin colour

Model 4: Model 3+ adjusted for marital status

*OR* odds ratio

**P < 0.01; *P < 0.05

## Discussion

The present longitudinal study confirmed the hypothesis that downward social mobility and experiencing low-income during adulthood increase the risk of worsening of oral health over time. Furthermore, the hypothesis that decrease on the social networks of family members and friends and persistent small stable number of social networks during adulthood predicts worsening of oral health was confirmed. Thus, enduring low income and small social networks seems to negatively influence SROH over time in adults.

Our findings support the social mobility hypothesis as one of the life course models in dental research that suggest that health inequalities are influenced by different social trajectories [4]. Overall, the present results are in accordance with previous longitudinal analysis on the impact of downward social mobility on oral health inequalities in adults [5–8]. However, there is no consensus on the influence of upward mobility and stable low social mobility on oral health during adulthood [8–10]. A recent systematic review on the influence of social mobility on tooth loss concluded that individuals in the upwardly mobile, downwardly mobile, and persistently low socioeconomic group were more likely to have tooth loss than those with persistent high social status [3]. Existing dental literature has also shown the relationship between income decrease over three years follow-up and poor SROH as well as the association of downward and stable low social mobility with number of teeth during adulthood [8, 10]. It is important to emphasize that oral health outcomes were assessed only at the end of the follow up period in the previous studies [8, 10]. The use of different oral health outcomes, different periods of follow up and distinct measures of socioeconomic position might also explain the discrepancies.

This study brings original evidence on the long-term importance of the number of social networks on adult’s oral health trajectories since participants experiencing decrease on social networks and persistent small social networks over the 13 years period of study reported worse SROH trajectories. Our findings may suggest that persistent small social networks could gradually accumulate over time and impact on self-rated oral health. This mechanism considers the amount and duration of exposures and proposes that ‘wear-and-tear’ adds up over time to affect health [21]. Previous cross-sectional studies showed the relationship between social networks and subjective oral health outcomes [11–14, 24], but others failed to report such association [25]. Similar to our findings, the number of close ties was associated with poor self-rated oral health among English adults aged 50 years or older [23]. Moreover, social network measures, including frequency of meeting friends and participation in sports and hobby clubs, was associated with self-reported number of teeth among elderly people [11, 12]. Social network of friends was also inversely associated with poor self-rated health in pregnant and postpartum women [14]. However, a study involving older American adults did not find association between social networks and self-rated oral health [25]. Methodological discrepancies, including study design, measurements of social networks and subjective oral health, and differences of demographic characteristics of participants, might explain the discrepancies between the study’s findings.

The present study has some limitations that should be considered. Using a single question to assess SROH may have resulted in an unspecific outcome measure. The adoption of multi-items questionnaires to assess SROH is recommended in future research since they are considered more sensitive measures. Social networks were assessed according to the participant’s perception on the number of family members and friends they had close social ties. Thus, the quality of the social networks was not considered in this study. In addition, nearly 30% of the participants in the wave 4 were excluded from the analysis due to missing data. Although the variables SROH and number of social networks did not differ between participants with missing data and the final analytic sample, the latter included a greater proportion of adults in the reference category (‘high-income-stable’ group) in the ‘income groups’. Thus, selection bias might have underestimated some of the reported associations between income groups and SROH.

This seems to be the first longitudinal study on social mobility and oral health involving a cohort of employed adults from one Brazilian university. Although this resulted in high retention rate of nearly 70% after 13 years, our findings should not be generalised to other populations. Most participants (85.6%) reported good-stable SROH, which may also impose some restrictions to extrapolate our findings. The possible reason for a high proportion of participants reporting stable SROH is the moderate and high the socioeconomic status of the studied sample, since nearly 73% and 76% of the participants reported per capita monthly income ≥ 3 Brazilian minimal wages (US$ 171.51/month) and 11 or more years of education at baseline, respectively [17]. A previous study using data from a nationally representative sample of adults living in Brazil showed that poor self-reported oral health measures were strongly associated with lower income and lower schooling [26].

The strengths of the present study were the assessment of income changes and social networks changes over more than one decade of follow up during adulthood and the evaluation of SROH trajectories in the same cohort. In addition, the regression models were fitted according to a theoretical model encompassing the study hypotheses [17]. Despite the above-mentioned critiques related to SROH measure used in this study, SROH is considered a comprehensive, reliable and valid measure in epidemiologic research that correlates with dental clinical measures [19]. In addition, self-perceived oral health measures capture the individual perceptions of subjective components related to health, including quality of life, well-being, and the oral impacts on physical and social functions [27].

The potential mechanisms by which income and number of social networks may influence adult’s perceived oral health over time include the behavioural and psychosocial explanations [28]. Individuals from lower socioeconomic groups and those with small social networks tend to engage in health-damaging oral health behaviours. For instance, social position and social ties indirectly predicted adult’s SROH via psychological distress and smoking when data from the present cohort was analysed [17]. Frequency of dental visits also mediated the link between social position and SROH in a previous study [17]. Yet, the relationship between social ties and SROH was not mediated by dental visits. A recent study revealed that young and middle-aged male adults with more close social ties were more likely to use preventive dental care [29]. The authors emphasized that social relationships may lead to higher compliance with health norms.

It is interesting to note some important variations in *per capita* family income and in the number of social networks over the 13 years period, despite the fact that this was an adult population at the same workplace throughout the study period. Around 30.1% and 11.8% of the participants experienced a decline in income and in the number of social networks, respectively. Another relevant aspect is the fact that nearly 25% of the participants were in the low income-stable group, and 12.1% of the sample reported small stable number of social networks at baseline and at 13-years follow-up. The influence of income changes and social networks changes on SROH draws attention and should prompt initiatives to tackle income-related oral health inequalities and to enhance adult’s social ties due to potential negative impact on physical, and mental health [30]. Different types of social network interventions have been proposed, including enhancing existing network linkages, developing new social network linkages, or enhancing networks through community capacity building and problem solving [31].

Future studies should consider longer follow up period to evaluate the effects of social mobility and social networks changes during working life as well as during retirement on people’s oral health. Although self-rated oral health is considered a valid and comprehensive measure of oral health status [19], future studies should combine subjective and dental clinical measures as outcomes.

## Conclusion

To the best of our knowledge, this is the first study that longitudinally evaluated the influence of income changes and changes in the number of social networks on SROH trajectories among adult workers. The present findings highlight the long-term influence of persistent poor income and downward social mobility on SROH trajectories during adulthood. Moreover, adults with a decrease on social networks of family members and friends, and those with small number of social networks over 13 years were at higher risk of reporting worse SROH during the study period. Possible strategies to tackle income-related health inequalities and to enhance the number of social networks from existing network linkages and/or developing new social networks should be considered to enhance adult’s oral health.

## Data Availability

The data that support the findings of this study are available from the Institute of Social Medicine, State University of Rio de Janeiro but restrictions apply to the availability of these data, which were used under license for the current study, and so are not publicly available. Data are however available from the authors upon reasonable request and with permission of the Institute of Social Medicine, State University of Rio de Janeiro.
